# Direct-fed Microbials (DFM) and Poultry Genomics: A Synergistic Approach to Sustainable Antibiotic Free Farming

**DOI:** 10.1007/s12602-025-10618-y

**Published:** 2025-06-14

**Authors:** Abdallah S. I. Hassan, Ahmed R. Henawy, Youssef A. Saied, Karen A. Garas, Omar M. Shahat, Asmaa A. Halema

**Affiliations:** 1 Animal Production Department, Faculty of Agriculture, Cairo University, Giza, 12613 Egypt; 2 National Key Laboratory of Agricultural Microbiology, College of Life Science and Technology, National Engineering Research Center of Microbial Pesticides, Huazhong Agricultural University, Wuhan, 430070 People’s Republic of China; 3 Department of Microbiology, Faculty of Agriculture, Cairo University, Giza, 12613 Egypt; 4 Department of Biotechnology, Faculty of Agriculture, Cairo University, Giza, 12613 Egypt; 5 Department of Genetics, Faculty of Agriculture, Cairo University, Giza, 12613 Egypt

**Keywords:** Poultry, Direct-fed microbials (DFMs), Multi-omics, Gut microbiome, Gut-brain axis

## Abstract

Improper usage of antibiotics in poultry production is a great threat to the ecosystem because their residues can enter into the food chain or leach into soil or water systems and increase antibiotic resistance risks. Hence, direct-fed microbials (DFMs) have gained recognition as a sustainable and viable alternative to antibiotics in poultry production, capitalizing on the relationship between microbial genetics, host genomics, and gut microbiota. This review delves into the genetic and host genomic mechanisms through DFMs effects including the enhancement of nutrient metabolism, modulation of gut microbiota and strengthening of the host immunity. The revolution of multi-omics has participated in the identification of probiotic strains with desirable traits and revealed their impact on host gene expression, particularly in genes related to intestinal health, such as tight junction proteins and mucins. Furthermore, the review summarizes the benefits of using DFMs in poultry production, the factors affecting their efficacy and their challenges and limitations. Future research integrating host and microbial genomics, along with precision microbiome engineering, holds promise for maximizing the potential of DFMs in advancing sustainable poultry farming practices.

## Introduction

**Fig. 1:**
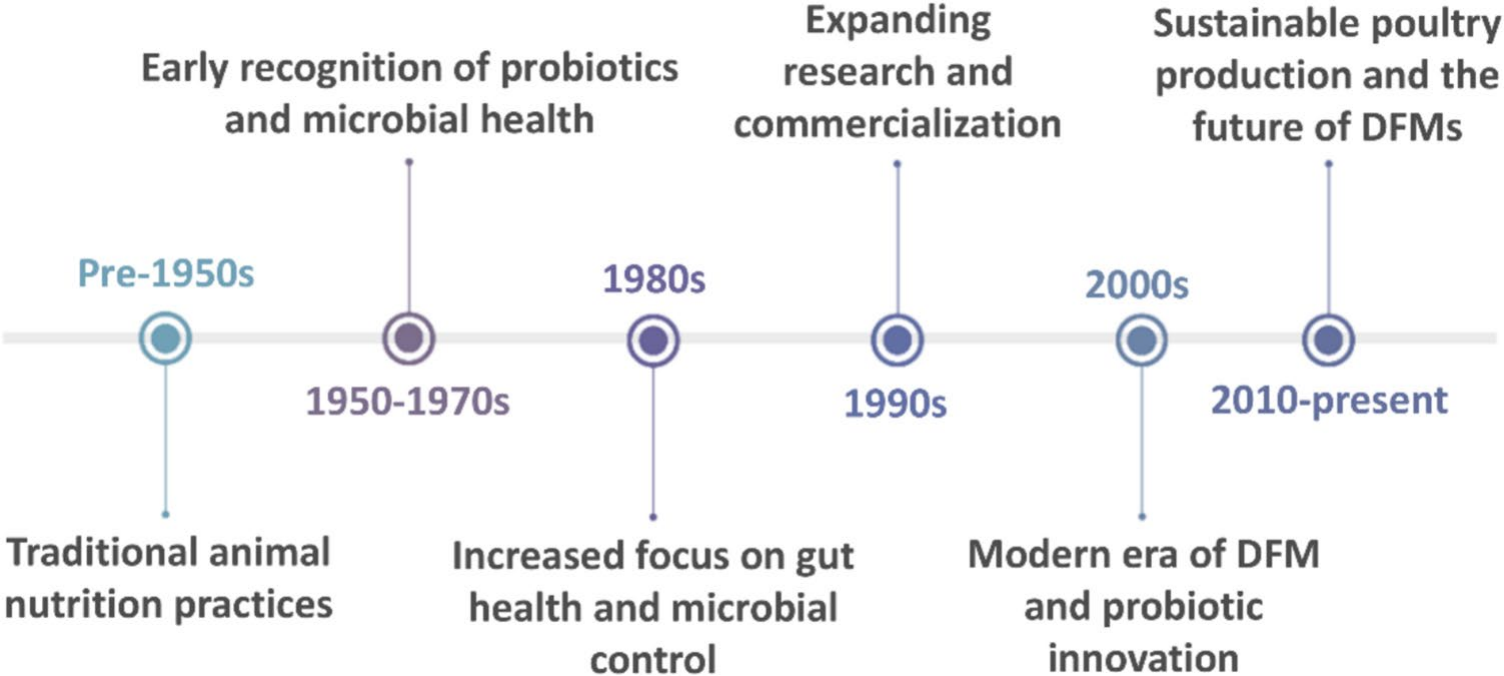
The historical context of the evolution of DFMs in animal nutrition, focusing on poultry

Beneficial living microorganisms can be supplemented in poultry feeds to improve digestive health which is called Direct-Fed Microbials (DFMs) or probiotics. Now, DFMs have become widespread in poultry production as a sustainable alternative to antibiotics. Their effects are reflected in nutritional consumption, growth rates, egg production, and intestinal health which are the subject of ongoing research [1]. DFMs have numerous roles focusing on gut health, immunological modulation, nutritional utilization, and pathogen control, in addition to decreasing antibiotics usage and minimizing environmental effects. Hence, they provide a sustainable and natural method to enhance animal welfare and productivity [2, 3]. During the past few decades, increasing the demand for sustainability in addition to advances in animal health and microbiology sciences participated in the development of using DFMs in the nutrition of animals, particularly in poultry (Fig. 1). Before the 1950 s, macronutrients and energy sources were the main factors in animal nutrition [4]. Neish [5] stated that not many people agreed that microbes might be involved in digestive health. During the 1950 s and 1960 s, antibiotics were first applied in animal feed to enhance growth and prevent diseases such as penicillin and chlortetracycline which became popular and widespread in poultry and livestock industries [6]. The probiotic term was first coined in 1965 by Lilly and Stillwell, referring to substances synthesized by one microorganism that facilitate the growth of another organism [7]. Early studies in the 1960 s and 1970 s started to explore the potential for beneficial microorganisms (bacteria, yeast) to enhance gut health and reduce intestinal diseases in poultry [8]. Since the late 1980 s, an enormous increase in interest in products depending on yeast and/or filamentous fungi which enhance gut functions as probiotics [9]. During the 1990 s, using of DFMs expanded [10] and the use of DFMs became more common in commercial poultry farming [6, 11]. By the early 2000 s, DFMs were widely accepted in many countries as safe and effective feed additives. The European Union, for example, started to regulate probiotics as a"zootechnical additive"to improve animal performance and health [12, 13]. From the 2010 s till now, the poultry industry has increasingly focused on sustainability [14, 15]. Furthermore, advances in multi-omics technology, including the sequencing of microbial genomes, metagenomics, nutrigenomics, proteomics and metabolomics, enabled more precise identification and enhancement of probiotic strains and studied their pathways. This led to the improvement of more effective and targeted probiotics for poultry [16–18]. This review proposes the importance of DFMs utilization as alternatives to antibiotics and focuses on their genetic mechanisms and effects on poultry.

## Types of Direct-fed Microbials (DFMs) Used in Poultry

The utilization of probiotics in poultry has exhibited a consistent increase yearly, attributable to the growing need for antibiotic-free poultry and the extensive body of research supporting their benefits. In 2023, the poultry probiotics market was valued at about 111.73 million dollars and is expected to elevate to 6.4% at a compound annual growth rate (CAGR) between 2024 to 2032 [19]. In poultry production, DFMs include numerous living microorganisms, i.e. bacteria, yeasts, and fungi, that preserve a healthy digestive system, herewith enhancing the poultry’s overall health and growth performance. These microorganisms can be categorized into types depending on their genus, species, and functional traits [1]. Probiotics for poultry are most frequently utilized from the following genera: *Bifidobacterium, Lactococcus, Lactobacillus, Bacillus, Streptococcus*, and yeasts like *Candida and Saccharomyces*. Each genus has distinct modes of action. For example, Lactic acid bacteria (LAB) produce lactic acid, which inhibits pathogen growth by reducing the gut pH [20], participating in overall gut health and enhancing the beneficial microbiota growth [21]. As well as *Bacillus* species are perfect for use in animal feed because of their strong resistance to environmental stressors such as heat and desiccation [22]. Furthermore, yeasts are frequently employed as DFMs by improving nutritional absorption and generating beneficial metabolites, such as B vitamins [23].

Since *Enterococcus* strains can withstand gut acidity (pH of 9.6) and preserve a balanced microbiota, they considerably exist in poultry probiotics [24, 25]. Additionally, the enhancement of *Bifidobacterium* species growth through the gut microbiota of the host that supplies carbon sources, which in turn aid in regulating pathogenic bacteria and sustaining a balanced intenstinal ecosystem [26]. Furthermore, fungi are utilized as DFMs in poultry production by elevating the colonization of beneficial bacteria, decreasing the colonization of enteric bacteria [27] and producing enzymes which enhance feed digestibility and disease resistance [28].

## Mechanisms of Action

**Fig. 2:**
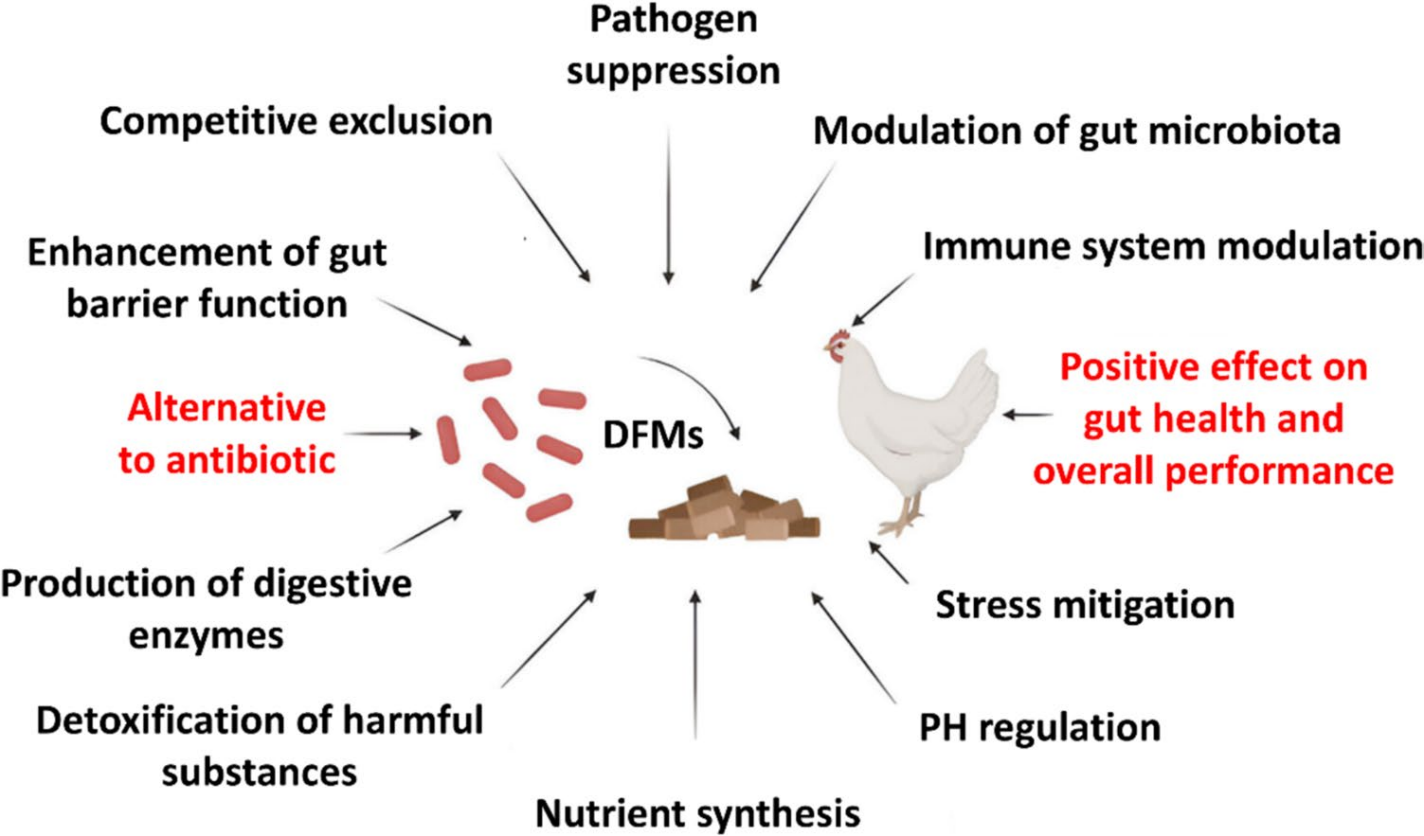
The mechanisms of action of DFMs used in poultry

DFMs have a variety of mechanisms of action that are inter-connected as shown in (Fig. 2). DFMs provide comprehensive benefits to poultry. Their ability to positively influence gut health and overall performance makes them a valuable alternative to antibiotics in sustainable poultry production systems [21].

Beneficial microbes in DFMs outcompete pathogenic microbes for attachment sites and nutrients in the gut. DFMs colonize gut surfaces, forming a protective barrier. They consume nutrients that would otherwise support pathogen growth like *Salmonella, Clostridium perfringens*, and *Escherichia coli* [29, 30]. Also, specific probiotic strains can provide metabolites and are characterized to decrease the numbers of *E. coli, Pseudomonas* spp. and *Salmonella* spp., through decreasing pH, antibacterial agents’ potentiation, and permeabilization of the cell membrane [31, 32]. Likewise, DFMs supply other antimicrobial compounds, including carbon dioxide, diacetyl, reuterin, bacteriocins, hydrogen peroxide, and acetaldehyde, which have been characterized to mitigate pathogens and have anti-inflammatory properties [33, 34]. DFMs strengthen the gut epithelial lining, preventing pathogen invasion and leakage of toxins [35]. Ban and Guan [36] reported that DFMs help establish and maintain a balanced gut microbiome. Notably, probiotics can increase the microbial enzymes and activate the host enzymes respectively [37]. Amylopectin is hydrolyzed by *Lactobacillus* species to produce glucose, maltose, and maltotriose [38]. Moreover, *Bacillus coagulans* NJ0516 increased the amylase activity in the broiler’s duodenum [39]. Cheng et al. [40] and Jha et al. [1] demonstrated that *B. licheniformis* and *L. bulgaricus* elevated the proteins, starch, and amino acids digestibility. Similarly, in infected hens with *Salmonella Typhimurium, Pediococcus acidilactici* conjugated with butyric acid and enhanced amylase activity [41]. Some DFMs synthesize essential nutrients that poultry cannot produce themselves. Consequently, the alternations in microbial populations within the gastrointestinal tract (GIT) induced by probiotics enhance the production of short-chain fatty acids (SCFAs) and facilitate immunomodulation, thereby improving energy metabolism as well [42]. Additionally, there are vitamins produced in the gut through different probiotics, including thiamin, B12, and folate [43]. Some DFMs can bind and neutralize harmful substances, i.e. heavy metals, or mycotoxins in the gut [44–46]. By decreasing cytokine production and preventing intestinal infections, a DFM may be incorporated during heat stress to decrease the stress response and boost overall performance and survivability [2].

## Benefits of Direct-fed Microbials (DFMs) in Poultry Production

DFMs offer a wide range of benefits in poultry production. Using DFMs has become an increasingly important part of poultry management. A high daily feed intake, and body weight gain (BWG) are often related to improvements in growth performance, meat quality, laying performance, health and disease resistance, stress reduction and antibiotic-free poultry products. Sarsour et al. [2] reported the significance of using DFMs in poultry production, noting that DFMs reduce cloacal temperature and panting behavior during heat stress in hot climates. These effects are linked to improved regulation of the hypothalamic–pituitary–adrenal axis, resulting in lower corticosterone levels in the blood, reduced stress responses, and increased ileal energy digestibility.

The incorporation of probiotics with broilers’ feed has been shown to boost production performance by increasing daily feed intake, calcium and nitrogen retention, and reducing the length of the intestine. The hypothesis that probiotics elevate the intestinal fermentation rate and stimulate SCFAs synthesis has been proposed, which serve as essential nutrients for intestinal epithelial cells, subsequently resulting in improved mineral assimilation [47]. Abd El‐Hack et al. [48] conducted a study examining the commercial multi-strain probiotics effects on performance and egg quality. The findings revealed that administration with probiotics led to significant improvements in various parameters associated with egg production, including the weight and size of the egg, albumin and yolk weight, as well as eggshell thickness and strength, in comparison to the control group.

Moreover, increased villus height, reduced crypt depth, and a higher villus height-to-crypt depth (VH:CD) ratio are key indicators of enhanced intestinal morphology, reflecting an expanded surface area that supports improved nutrient absorption. Similarly, the goblet cell numbers present in the intestinal villi and crypts act as another marker of intestine health, due to these cells being involved in mucin production and prevent the adhesion of harmful pathogens to the intestinal epithelium [49]. The supplementation of *Lactobacillus salivarius* and *Lactobacillus reuteri* [50], *Pediococcus acidilactici* [41], *Lactobacillus acidophilus*, *Lactobacillus casei*, *Enterococcus faecium* and *Bifidobacterium thermophilum* mixture [51], *Bacillus subtilis*, *Bacillus licheniformis,* and *Saccharomyces cerevisiae* mixture [52], *Bacilluscoagulans* [53], and *Propionibacterium acidipropionici* [54] were examined in laying hens to record the histomorphological alterations induced by probiotics. These results showed a positive effect on the histomorphological parameters of the small intestinal villi, proved by boosting both villus height and the ratio of villus height to crypt depth, which is related to boosting intestinal nutrient uptake and improving intestinal architecture. Meat quality can be improved by probiotics. *Saccharomyces cerevisiae* has been demonstrated to have the ability to raise ghrelin, as well as support the metabolism and absorption of fatty acids, maintain glucose uptake [55], and increase the levels of muscular calcium [56, 57], all of which improve the softness of the meat. Huang et al. [58], put out a different explanation, arguing that calcium ions in muscles regulate calpain activity and modify the kind of muscle fiber, which impacts meat quality and enhances fat and metabolism. The enhancement of protein deposition and muscle fat affects the flavor, water and cooking loss, and tenderness of meat [21]. Furthermore, using DFMs provides us with antibiotic-free poultry production [59]. Additionally, the reduction of mortality of poultry fed diet containing DFMs and better overall profits on returns from their production, so it is more economical to rear poultry on DFMs diet [60].

## Factors Affecting the Efficacy of Direct-fed Microbials (DFMs)

Although some studies highlighted the beneficial effects of probiotics supplementation on poultry production, others failed to confirm these positive effects [61]. The existence of contradictory results can be attributed to numerous factors such as the certain probiotic strain used, the implemented hygienic measures [61], the probiotic dosage, and also the age, breed, and poultry species, the inoculation level of threatening pathogens, and other various external factors [1]. Many studies were carried out to identify the optimal probiotic dosage and its potential advantages. However, setting definitive recommendations remains an obstacle due to the differences in dietary practices, and stress levels noticed in various settings. More studies are required to identify the ultimate dosage of single- or multi-strain probiotics and to assess their effects in birds exhibiting intestinal disorders and compromised gut health [1]. Each strain has its own preventive effect so many commercial products have multi-strain probiotics [1]. Multi-species probiotics have synergistic effects by operating on distinct areas and having various action modes [62–64]. The basic criteria for selecting probiotic strains lie in adherence ability to the gastrointestinal mucosa, gastrointestinal conditions tolerance, and the pathogen's competitive exclusion [65]. Additionally, their capability to withstand the conditions of manufacturing, transportation, storage, and application processes, as well as their overall viability are important selection criteria [66]. Furthermore, the climate is a critical factor that affects poultry performance and alters the gut microbiota. Amplicon metagenome studies have reported that poultry became more susceptible to *E. coli* infections and enhanced the colonization of the intestine by *Salmonella* according to climatic conditions [67]. Farm climate depending on geographic location significantly affects the intestinal microbiota. Unfavorable environmental conditions can lead to reduced poultry production, highlighting the significance of climate-related challenges management to maintain poultry health and performance [67]. Although the strain of microorganisms was used, environmental factors such as the intensity and length of heat exposure, relative humidity, size, and the age of the broilers could all affect this reaction, DFM administration may change HS responses [2].

The immune system and gastrointestinal tract development are impacted by the comparatively high levels of sanitation in poultry production. Due to bacterial exposure from different environmental sources, i.e. transport crates, people handling the chicks, initial feed, and litter inside the poultry house, the high hygiene levels produce a variable intestinal microbiota. This intestinal bacterial community is formed in a rather random manner and exhibits significant heterogeneity. The intestinal morphology of chickens kept in isolators changed, showing shorter villi, more shallow crypts, and less formation of acidic mucus, which could result in a changing composition of the microbiota [67].

The cage effect in poultry is linked to their coprophagic behavior, where they consume feces, leading to the uncontrolled intake of particles and feathers that can influence the intestinal microbiota. Poultry raised on organic farms show higher levels of *Clostridium perfringens* in the ileum and cecum compared to those on conventional farms. This discrepancy might result from conventional feed’s usage of salinomycin, an antibiotic coccidiostat that modifies microbial composition. Organic poultry also exhibits lower *Enterobacteriaceae* levels and higher Lactobacilli in the ileum, while free-range poultry shows a greater abundance of bacteria linked to amino acid and glycan metabolism in the ceca. The flock's collective microbiota significantly impacts individual poultry performance and gut bacterial communities [67].

## The Genetic Basis of Host-Microbe Interactions

Among all birds, chickens constitute the major important economic value all over the world through a global production of eggs by around 87 million metric tons in 2022. As well as, by 2024, global meat production is predicted to grow to around 104.2 million metric tons [68]. So, it is necessary to study its genetic makeup and variations of its mutant phenotypes that led to different characteristics including performance, egg weight, mortality, carcass weight and growth rate.

In 2003, the chicken genome started at Washington University which consisted of ∼1.2 billion-bp. In 2004, the deposition of the first draft genome sequence was carried out into free public databases. A female red junglefowl *G. g. gallus* was the selected bird for genome sequencing, the wild ancestor to the domestic chicken *G. gallus domesticus* and an estimated 20,000–23,000 genes arranged in 39 pairs of chromosomes which are the origin of hereditary divided into 38 pairs of autosomes that show distinctive features of poultry, while only one pair of sex chromosome (Z and W) that determine the gender of offspring either female or male [69]. This draft genome sequence offers valuable insights into vertebrate genome evolution and contributes to the enhancement of mammalian genome annotation, genome architecture and molecular evolution. Moreover, the chicken serves as the main bird model in the lab and is an essential model system for developmental biology research as well as studies pertaining to immunology, virology and oncogenesis. Additionally, the genome sequencing of the chicken will make it easier to map quantitative trait loci related to this important agricultural animal [70]. Hence, large-scale genome research has gained prominence. Consequently, a study conducted as part of the Genome-Wide Association Study (GWAS) identified numerous single nucleotide polymorphisms (SNP) [71, 72]. As well as several regions associated with selection signatures were also detected, indicating that these loci in native chicken breeds may be utilized to pinpoint the causative genes and mutations involved in enhancing the related traits of meat and egg [73].

It is interesting to know how host genes affect DFMs which are not clearly enough. The relationship of host genetics with the structure of the microbiota community in their gut indicates that specific genes control the existence of different species of microbes such as certain species of Methanobacterium are strongly associated with the two genetic loci (rs15142709 and rs15142674). [74].The lck/yes-related novel tyrosine kinase (LYN) and pleiomorphic adenoma gene 1 (PLAG1), which are both involved in cell growth and differentiation, contain these loci. Further supporting the diversity in the community structure of the gut microbiota by about 21% is the locus rs16775833, which is found within the double sex and mab-3-related transcription factor (DMRT) gene. These findings have suggested that host genetics influences the gut microbiota's structure in birds, and it is vital to investigate its various functions and effects on poultry [74].

During the DFMs administration to food-producing animals, the host genetic factors and interactions between the host and environmental factors should be taken into consideration. It was proposed both that environmental and host genetic factors may determine the GIT microbiome individuality [75, 76]. Contemporary results have shown the impact of host genetic factors on individualized rumen microbiomes in cattle [76–78]. Conversely, the GIT microbial community was shown to be primarily unaffected by host genetics, as evidenced by SNPs in chickens [79]. However, heritable taxa were noted, some of which are linked to SNPs in cattle [80, 81]. Hence, more thorough research is needed to determine how environmental factors (like diet and barn) and genetic factors (like sex and breed) contributed to the variations in the GIT microbiomes of individual hosts and how these variations impact the efficacy of DMF use in food-producing animals [82].

Moreover, Ma and Guan [82] suggested that the structure and the core/individualized microbiomes function and how host genetic factors influence the use of DFMs are still not fully understood. Thus, more studies should be made to examine how, in food-producing animals, the GIT microbiome interacts with administrated DFMs in a host-specific or strain-specific way. Additionally, the genetic makeup of DFMs defines their ability to survive, colonize, and positively impact the poultry gut microbiome. By leveraging genomic insights, researchers can design more effective, targeted DFMs, contributing to sustainable and efficient poultry production systems [36]. As mentioned before, Bacteria are the most frequently used DFMs due to their diverse metabolic and functional capabilities [83] such as LAB and *Bacillus* species. The *Lactobacillus* core and pan-genome contain 266 genes and 20,800 genes respectively, while the average genome size and G + C content were 1.96 Mb and 37.2% respectively [84, 85]. On the other hand, *Bacillus subtilis* genome is composed of 4,214,810 base pairs, especially 4,100 protein-coding genes with an average G + C ratio is 43.5%, however, this varies significantly across the chromosome [86]. Some of the genetic interactions of *Lactobacillus* species with the host genome focus on certain functions including active metabolism, stress resistance, probiotic actions and adhesion [87] as summarized in Table 1).

**Table 1:** Exploring the genetic network: *lactobacillus* species and their gene-level interactions

Species name	Genes involved	Certain function	References
***L. reuteri***	*Inu, gtfA, xylA, met*	Active metabolism (Carbohydrate and protein metabolism)	[88–90]
	*lr1516, dltA, dps, clpL, clpE, msrB, Lr1265, Lr1584*	Stress resistance (Cell envelope, Protection, repair DNA and proteins, Active removal of stressors)	[88, 89, 91–94]
	*luxS*	Probiotic actions (Antipathogenic effects)	[95, 96]
	*lsp*	Adhesion (Cell surface proteins)	[89]
***L. johnsonii***	*LJ1654-LJ1656, prtP (LJ1840)*	Active metabolism (Carbohydrate and protein metabolism)	[97]
	*LJ0056, LJ1147, LJ1413*	Stress resistance (Active removal of stressors)	[97]
	*LJ1680*	Probiotic actions (Immunomodulation)	[97]
	*LJ1476*	Adhesion (Cell surface proteins)	[97]
***L. plantarum***	*pts14 C*	Active metabolism (Carbohydrate metabolism)	[98, 99]
	*clpC, copA, bsh1*	Stress resistance (Protection, repair DNA and proteins, Active removal of stressors)	[98–100]
	*dltB*	Probiotic actions (Immunomodulation)	[101, 102]
	*srtA, msa, lp_2940, lp_1403*	Adhesion (Cell surface proteins)	[98, 99, 103]
***L. acidophilus***	*bfrA, msmE, treC*	Active metabolism (Carbohydrate metabolism)	[104, 105]
	*LBA1272, slpA, cdpA, LBA1524, LBA1430, LBA1431, LBA1432* *gadC (LBA0057), LBA0867, LBA0995, LBA0996, LBA1427, LBA1428, LBA1429, bshA, bshB*	Stress resistance (Cell envelope, 2 CRS regulators, Active removal of stressors)	[106–111]
	*labT*	Probiotic actions (Antipathogenic effects)	[112]
	*fbpA, mub, slpA, cdpA, LBA1663- LBA1664*	Adhesion (Cell surface proteins)	[108, 113]
***L. paracasei***	*fosE*	Active metabolism (Carbohydrate metabolism)	[114]
***L. rhamnosus***	*dltD, luxS*	Stress resistance (Cell envelope, Protection, repair DNA and proteins)	[87, 115]
	*dltD*	Probiotic actions (Immunomodulation)	[115]
***L. sakei***	*rrp-1, rrp-48*	Stress resistance (2 CRS regulators)	[116]
***L. casei Shirota***	*cps1 A-J*	Probiotic actions (Immunomodulation)	[117]
***L. salivarius***	*abpT*	Probiotic actions (Antipathogenic effects)	[118]
	*srtA, lspA, lspB, lspD*	Adhesion (Cell surface proteins)	[119]

### Coevolution Between Direct-fed Microbials (DFMs) and Host

Coevolution refers to the reciprocal evolutionary adaptations between interacting species over time [120]. The relationship between *Lactobacillus johnsonii* and its host, including chickens (*Gallus gallus domesticus*) and turkeys (*Meleagris gallopavo domesticus*)) represents a fascinating example of coevolution, where this probiotic bacterium has adapted to thrive in the host's gastrointestinal environment, while the host benefits from the bacterium’s contributions to health and fitness.

However, a growing body of evidence indicates that using host-specific strains could be essential, as their coevolution with the host animal enhances the strains'ability to establish and thrive inside its corresponding host animal species. This idea has been implemented in *Lactobacillus johnsonii* in commercial poultry farming because of its prior linkage to improved poultry performance [121]. Previous research showed the concept of using host-adapted microorganisms for DFM application in poultry, such as *Lactobacillus johnsonii*. Particularly, studies demonstrated that an *L. johnsonii*-based DFM derived from turkeys might improve early turkey poult performance by positively influencing the gut microbiome [122]. Johnson et al., [121] highlighted that host-adapted DFMs could imitate some of the beneficial effects and microbiome-boosting impacts induced by antibiotics used for growth enhancement. Similar research has been conducted utilizing a chicken-adapted *L. johnsonii* strain in broiler chickens [123]. Previous research indicated that the coevolution happens between *Lactobacilli* and their hosts [124], which can have a vital role in their next colonization and accommodation inside various hosts. However, choosing a suitable strain with probiotic potential remains important, even within bacterial species and host animal sources. Differences in the strain level present regarding phenotypic traits such as the ability to inhibit pathogens and withstand the conditions of gastrointestinal transit [125]. Ideally, a DFM candidate aimed at enhancing early poultry performance and supporting gut microbiome establishment should be specifically adapted to the host in addition to possessing and exhibiting some of the positive phenotypes associated with an effective probiotic.

Notably, an examination of the core SNP distances among the various isolate groups provided additional evidence in favor of the theory that *L. johnsonii* and its host coadapt. This corroborates the concept that the host and *L. johnsonii* coevolved [126]. The animals’ immune system evolution has been attributed to *Lactobacilli,* which seems to coevolve with their host while maintaining an association that prevents an overabundance of immunological response [127]. Genetically, Johnson et al. [121] proved that *L. johnsonii* clusters phylogenetically by host. Even somewhat closely related host species are affected by this. Nevertheless, the combination of compelling in vivo data with robust genomic and in vitro data indicates that *L. johnsonii* coadapted between poultry host sources and the host-specific strains of the parasite may have a greater and more enduring effect than non-host-specific strains.

### Gene Regulation and Expression in Microbiota

DFMs influence gene expression within the poultry gut microbiota through metabolite production, competitive interactions, and immune modulation. By reshaping microbial transcriptional profiles, DFMs contribute to gut health, pathogen resistance, and improved nutrient absorption.

Chichlowski et al. [51] found how the entrance of DFMs could shape the poultry’s GIT and affect its microarchitecture through ileal, jejunum, cecal, and colonic epithelium. The results demonstrate that DFM administration caused a significant increase in intestinal muscle thickness, with an enhancement of up to 33% compared to the control treatment. Additionally, the jejunum's villus height and perimeter were significantly greater in the group treated with DFM. Furthermore, this DFM-treated group exhibited elevated values for muscle thickness and jejunal crypt depth. Similar improvements in muscle thickness were observed in the ileum of birds that received DFM. Conversely, the DFM-treated groups demonstrated reduced densities of bacteria embedded within the mucous blanket and a decrease in mucous thickness across all intestinal segments in contrast to the control.

The influence of a multi-strain *Bacillus*-based DFMs (*B. licheniformis*, *B. amyloliquefaciens*, and *B. subtilis*) combined with fenugreek seed on the expression of specific immune genes through upregulation and downregulation mechanisms. All studied genes (AvBD (avian ß-defensins), PTGS2 (prostaglandin-endoperoxide synthase 2), IL6 and IL8L2 (pro-inflammatory interleukins), CASP6 (caspase), and IRF7 (interferon regulatory factor 7)) were downregulated except AvBD10 gene was upregulated [3]. These results suppose that both transcription factors (FSs) and DFMs may alleviate inflammation and improve broiler immunity.

Additionally, it has been demonstrated that using *Bacillus licheniformis* as a DFM greatly increases the gene expression linked to catabolism in the livers of afflicted organisms, particularly carnitine palmitoyltransferase-1 and peroxisome proliferator-activated receptor-α. Furthermore, this intervention altered the gene expression associated with lipid anabolism. These results suggest that the supplementation with *B. licheniformis* in diet may improve growth and antioxidant capacity while also modulating the gene expression responsible for the synthesis of fatty acid and oxidation in the broiler livers diseased with Necrotic Enteritis (NE) [128].

## Genetic Mechanisms of Direct-fed Microbials (DFMs) in Modulating Host Gene Expression

### Immune System Modulation

Poultry immune system components can be categorized into innate and adaptive immune responses and considered complex mechanisms which protect them from any infection [129]. Lee et al. [130] stated that DFMs may directly affect the gut microbiota composition, which is critical for the immune system development and maintaining homeostasis. This includes antimicrobial peptides (AMPs), toll-like receptors (TLRs) and cytokines in chickens and mammals.

TLRs are critical pattern recognition receptors (PRRs) in chicken, expressed in the intestine and crucial for microbial-associated molecular patterns (MAMPs) identification from bacteria and viruses [131]. Additionally, they initiate innate immunity and activate adaptive responses through initiating signaling pathways that stimulate T cells and cause pro-inflammatory cytokines to be released [132, 133].

DFMs produce MAMPs that can be recognized by TLRs on dendritic cells in the gastrointestinal lumen [134]. Numerous types of TLRs are distinguished in poultry i.e. TLR2 (paired with TLR1) for detecting peptidoglycans and lipoproteins in gram-positive bacteria [135], TLR4 for recognizing lipopolysaccharides (LPS) from gram-negative bacteria, TLR5 for identifying bacterial flagellin [136], TLR15 for detecting bacterial proteases and heat-stable secretory substances [137], TLR3 and TLR7 for recognizing double-stranded and single-stranded RNA viruses, respectively [138], and TLR21 for identifying unmethylated CpG-oligodeoxynucleotides from bacteria and viruses [139]. When TLRs are activated, the expression of AMPs, cytokines and innate immunity will be regulated in the intestine of chicken [140, 141].

Cytokines adjust immune cell stimulation and various physiological processes [142]. They participate in precursor cell development, apoptosis, cell survival, tumor rejection, metastasis, and inflammatory responses [143]. Cytokines are critical for host growth, injury response, homeostasis, and infectious disease management, where they protect from pathogenesis [144]. Cytokines bind high-affinity receptors on target cells to initiate intracellular signaling with high potency [145]. According to their functions and cell interactions, cytokines are categorized into groups like tumor necrosis factors (TNF), chemokines, transforming growth factors (TGF), interleukins (IL), and interferons (IFN) with notable functional overlaps. Infectious agents, endotoxins, inflammatory mediators, injuries, or other cytokines stimulate their production [146].

Small peptides with 15–100 amino acids, known as AMPs, are essential for innate immunity because they prevent pathogen invasion [147]. Poultry performance can be improved by their extensive antimicrobial activities, which include antibacterial, antifungal, and antiviral actions [148–150]. Defensins and cathelicidins, two important AMP kinds, exhibit activity against enveloped viruses, fungi, and bacteria [151]. The innate immunological defense of chickens is greatly aided by the identification of four cathelicidins (CATH1-3, CATH-1B) and fourteen avian β-defensins (AvBD1-14). These peptides fight infections, which enhance poultry productivity and health [140, 152–154]. Many studies used various types of DFMs to investigate their effect on toll-like receptors, cytokines, and antimicrobial peptides in *Lactobacillus* spp. and *Bacillus* spp. as a part of the immune system in poultry as proposed in Table 2).

**Table 2:** Modulatory effects of direct-fed microbials (DFMs) on immune-related gene expression

DFMs	Animal	Inclusion level	Duration	Response	Ref
**TLRs expression change**					
**Multi-strains (*Enterococcus feacium*,** ***Lactobacillus casei,*** ***Lactobacillus acidophilus,*** ***Bifidobacterium thermophilum*)**	broiler breeder hens	2.5 × 10^7^ cfu/g For each strain respectively	10 weeks	Increase in TLR2 and TLR4 mRNA expression	[155]
**Single strain** **(*Pediococcus acidilactici*)**	broiler breeder hens	1 × 10^10^ CFU/g	10 weeks	Increase in TLR2 and TLR4 mRNA expression	
***Lactobacillus acidophilus* strain (LA-5)**	chickens	1 × 10^9^ CFU/g	14 days	Increase in TLR2,TLR4 and TLR5 mRNA expression	[134]
***Lactobacillus acidophilus* strain (LA-5)**	chickens	10^9^ CFU/g	21 days	No effect on the TLR2,TLR4 and TLR5 mRNA expression	
***Lactobacillus reuteri* (*L*. *reuteri*)**	Broiler chicks	2** × **10^9^ CFU/g	7 days	Significant increase in the expression of TLR5	[141]
***Clostridium*** ***Butyricum* (*C. butyricum*)**	Broiler chicks	1.3** × **10^7^ CFU/g	7 days	Significant increase in the expression of *TLR2-1* *TLR5*	
**Cytokinins expression change**					
***L*. *reuteri***	Broiler chicks	2** × **10^9^ CFU/g	7 days	Increase in the expression levels o*−1β*, *TGFβ3* and *TGFβ4*	[141]
***C. butyricum***	Broiler chicks	1.3** × **10^7^ CFU/g	7 days	Increase in the expression levels of *IL-1β*, *TGFβ2* and *TGFβ4*	
***B. subtilis* strain 1781**	broiler chicks	1.5 × 10^5^ CFU/g	14 days	No differences in the expression of IL1β, IL17 F, increase in IL6 and TNFSF15	[65]
***B. subtilis* strain 1104 + strain 747**	broiler chicks	each strain represents 7.5 × 10^4^ CFU/g	14 days	No differences in the expression of IL1β, IL17 F, increase in IL6, IL8 and TNFSF15	
***B. subtilis* strain 1781 + strain 747**	broiler chicks	each strain represents 7.5 × 10^4^ CFU/g	14 days	No differences in the expression of IL1β, IL17 F while increase in TNFSF15	
**AMPs expression change**					
***L*. *reuteri***	Broiler chicks	2** × **10^9^ CFU/g	7 days	Significant Increase in the expression levels of *AvBD4* in the cecum	[141]
***C. butyricum***	Broiler chicks	1.3** × **10^7^ CFU/g	7 days	Significant Increase in the expression levels of *AvBD1* and *CATH3* in the cecum	
***Lactobacillus acidophilus, Bifidobacterium bifidum, and Enterococcus faecalis***	Broiler chicks	1** × **10^6^ CFU/g	5 days	No significant effect on the expression level of *AvBD1, AvBD2, AvBD4, AvBD6,* and cathelicidin	[156]

### Gene Expression in Intestinal Integrity

**Fig. 3:**
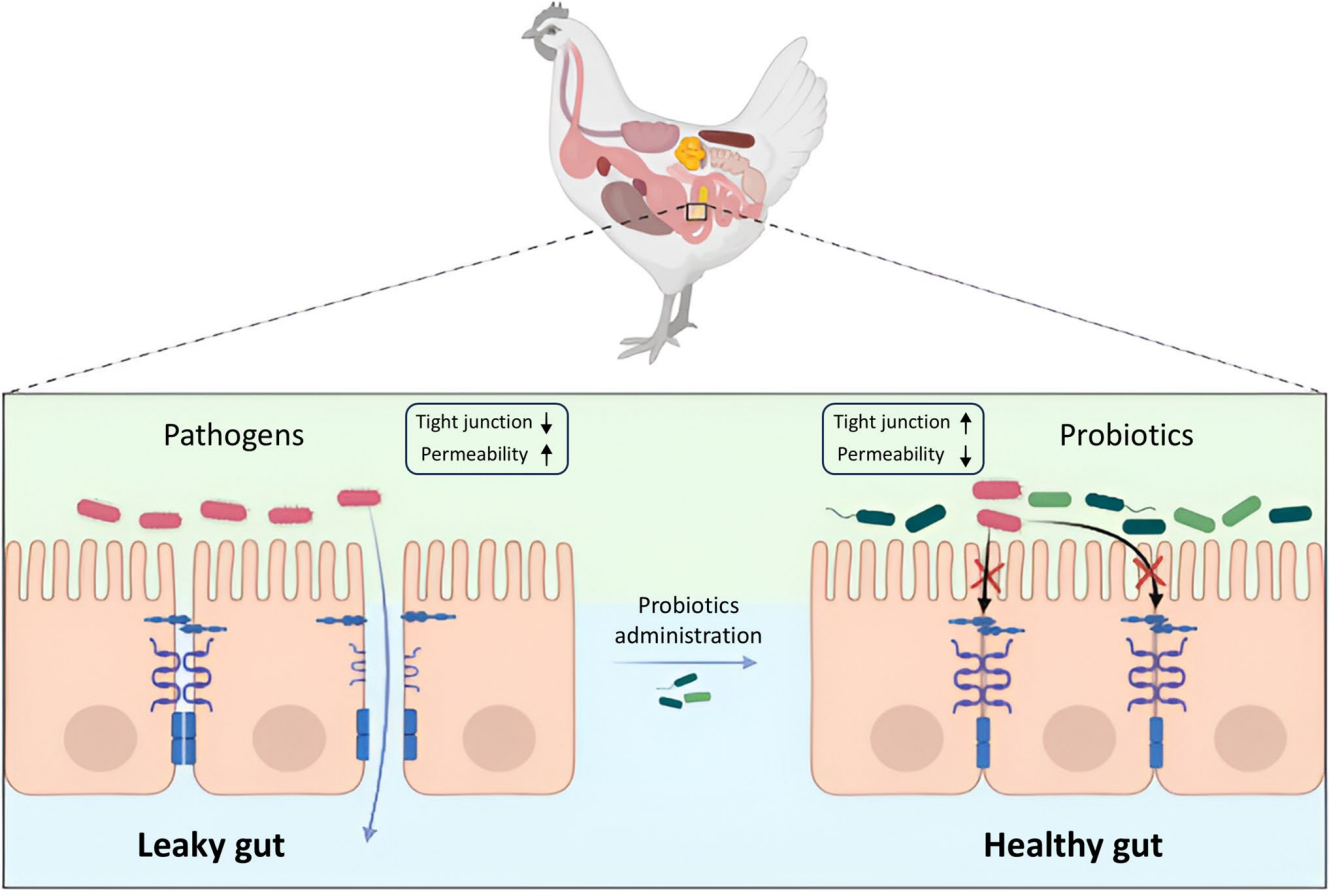
The impact of probiotics administration on intestinal barrier integrity

The intestinal barrier integrity is crucial for optimal health and performance in poultry as provided in (Fig. 3). It protects the gut from pathogens and harmful substances penetration however it allows the absorption of nutrients [157]. It consists of 3 structures: the first barrier is a physical barrier (intestinal epithelial cells, tight junctions (TJs), and mucin), the second is the chemical layer that includes (cytokines, immune cells, digestive secretions, and cytokines), and the third, modulation by gastrointestinal tract microbiota [158]. In the physical barrier, the goblet cells secrete mucin 2 (MUC2) which is the most common type of mucin, that works as the first line of defense against pathogenic bacteria [159]. The intestinal epithelium is an integral component of the gastrointestinal tract and consists of a single layer of column-shaped epithelial cells that act as a physical barrier against intraluminal toxins and invaders [160]. Intercellular junctional components give these epithelial cells their tight seal, allowing cells to adhere to one another and controlling paracellular permeability [65]. Many stressors can disrupt tight junctions [161], and cause gut leakage [162]. Heat stress, feed limitation for 24 h, high rye addition to broiler chicken diet, and high mycotoxin dose in the diet are some examples of these stressors [163–168].

Tight junctions are made up of structural and functional proteins i.e. zonula occludens 1 (ZO-1), Claudin (CLDN), Junctional Adhesion Molecule (JAM), and occluding (OCLN). When the tight junctions and the epithelial barrier are disrupted, a leaky gut will occur and allow pathogens to enter the gut and release toxic substances to the bloodstream, then lead to inflammation and other health issues [169, 170]. Several studies obtained that the promotion of intestinal barrier function will be carried out when the expression levels of intestinal barrier genes upregulate through probiotic administration [159].

Wu et al. [159] examined the nutritional supplementation effects on chicken broilers under controlled conditions i.e. from 32 °C to 26 °C at a rate of 2 °C per week. This experiment had three groups (the control group and two experimental groups were supplemented with 10⁸ CFU/kg of feed with either *Lactobacillus plantarum* 16 (Lac16) or *Paenibacillus polymyxa* 10 (BSC10). In BSC10 group, tight junction protein-related genes were upregulated, such as *occludin (OCLN), Zonula occludens-1 (ZO-1),* and *claudin-1 (CLDN1)*, as well as *mucin-2 (MUC2)* expression compared to the control group. Moreover, Gadde et al. [65] estimated the effects of different dietary treatments divided into five groups studied on zero-day-old male ross broiler chicks and divided them into five groups to evaluate the effects of different dietary treatments as the following: control group, a group receiving a basal diet with bacitracin methylene disalicylate (BMD) at 50 g/ton, and three groups supplemented with different *Bacillus subtilis* probiotics—strain 1781 (PB1), a combination of strains 1104 and 747 (PB2), and a combination of strains 1781 and 747 (PB3). Occludin expression was increased in the PB1 and PB2 groups, whereas JAM2 and (ZO1) were considerably upregulated in the PB2 and PB3 groups compared to controls. These findings indicated that probiotics influenced the tight junction proteins gene expressions. On the other hand, MUC2 expression was unaffected by any of the treatments.

Furthermore, all three dosages of *Bacillus licheniformis* markedly elevated the gene expression of CLDN-1, however, the claudin 2 (CLDN-2) levels were only up-regulated by 0.03% *B. licheniformis*. Although 0.01 and 0.03% *B. licheniformis* did not show any evident impacts on OCLIN-1 and ZO-1, 0.06% *B. licheniformis* significantly increased their expression level. Moreover, MUC2 expressions were markedly upregulated by *B. licheniformis* at three dosages. Generally, all the tested tight junction transcript levels are increased by *B. licheniformis*, and this effect is more noticeable at higher dosages of the bacterium [37]. Generally, chickens fed probiotic-supplemented diets showed an increase in TJ proteins, which translates to improving the function of intestinal barriers and optimal gut health.

### Gut-brain Axis and Neurotransmitter Regulation

**Fig. 4:**
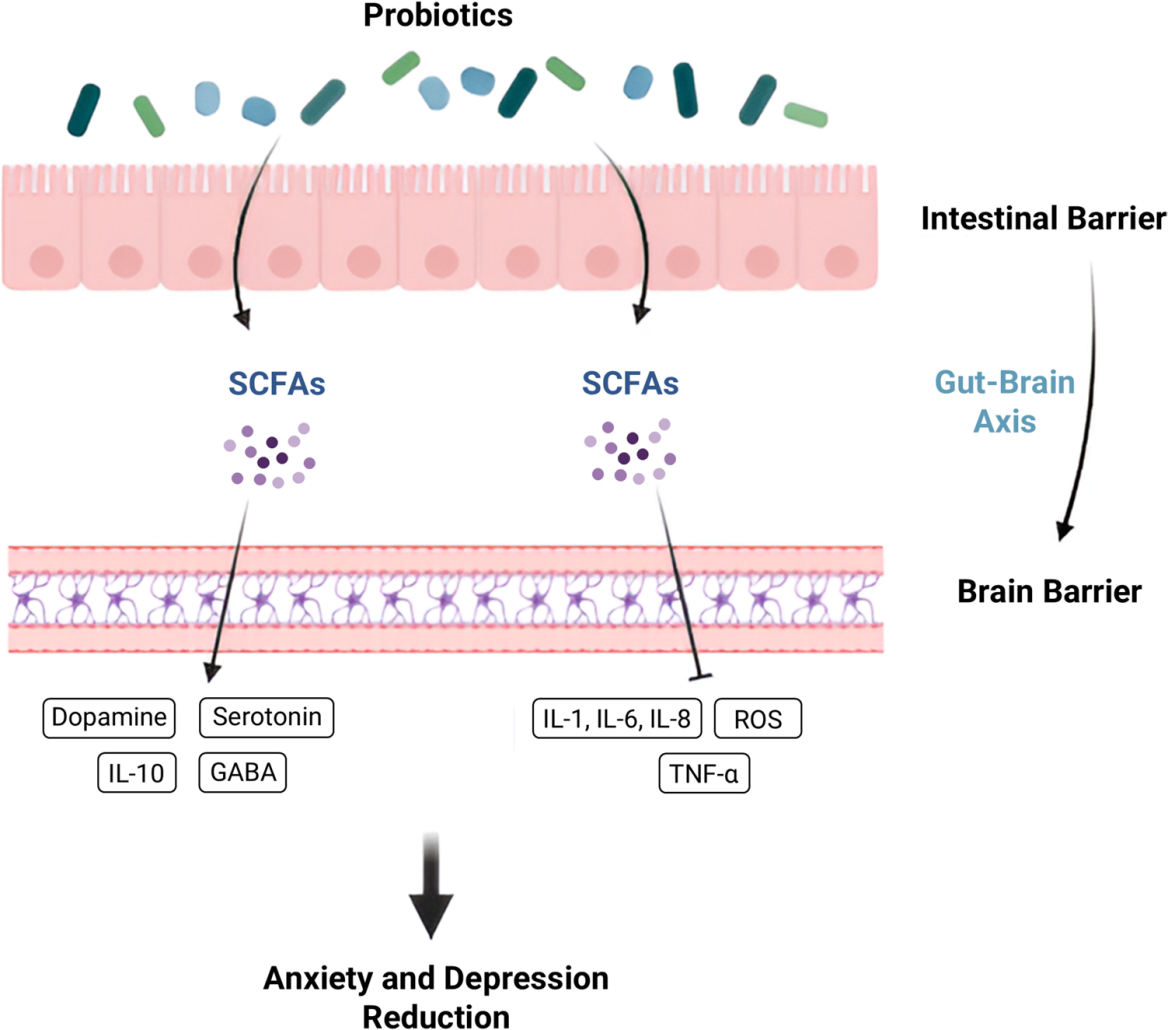
The gut-brain axis and the mechanisms of action of probiotic supplementation in order to alleviate anxiety and depression

A network of bidirectional communication between the central nervous system (CNS) and the gastrointestinal (GI) is known as the gut-brain axis. Via this intricate process, neural, immunological, and hormonal signals visceral messages, which can be produced by the host or gut bacteria, can be transmitted from the gut to the brain thanks to this complex mechanism. The signals from these messages were received by the brain through interaction with the enteric nervous system. Afterwards, the brain transmits signals that impact immune responses and other gastrointestinal processes, such as motor, sensory, and secretory activities. This reciprocal relationship causes local pathological circumstances and shapes gut physiology [171, 172]. Numerous studies explored the mechanisms by which probiotics affect the gut-brain axis. Probiotics directly influence the biochemistry of the central nervous system, affecting neurotransmitter levels such as dopamine, γ-aminobutyric acid (GABA), brain-derived neurotrophic factor (BDNF), and serotonin, which in turn affects behavior and mental health. Wang et al. [173] and Heidarzadeh Rad [174] reported that the vagus nerve and enteric nerves are connected. Indirectly, DFMs can enhance the central nervous system function through gut microbiota modulation. By raising the diversity of microbiota and the abundance of beneficial bacteria, probiotics can alter metabolites like short-chain fatty acids and tryptophan, which can positively impact brain function [14, 173]. Reducing anxiety and depression is considered a turning point in poultry as growth, plumage condition, egg production, eggshell quality, and the efficiency of food conversion have a negative correlation with high levels of underlying fearfulness [175]. Some bacteria such as (*Lactobacillus plantarum, Bifidobacterium lactis, Lactobacillus reuteri, Lactobacillus helveticus, Bifidobacterium bifidum, Bifidobacterium longum*) produce SCFAs induce the release of γ-aminobutyric acid (GABA), dopamine, Noradrenaline and serotonin (5-HT), while resulting in a reduction of pro-inflammatory mediators like TNF α, IL, IFN γ, and reactive oxygen species (ROS), these pathways can mitigate anxiety and depression [176, 177] as shown in (Fig. 4).

**Fig. 5:**
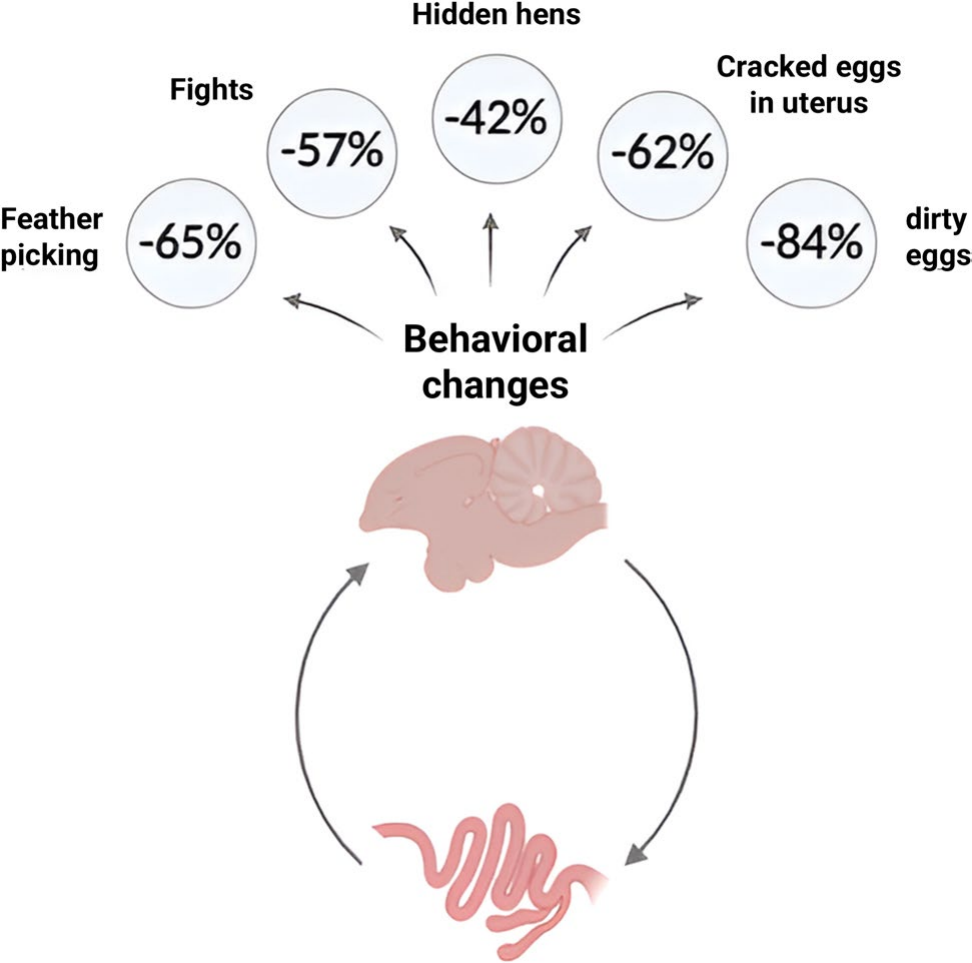
Impact of probiotics in gut-brain axis modulation and laying hen behavior

Noteworthy, probiotic use had a positive impact on indicators such as decreased rates of aggressive behavior, feather plucking, fights, concealed chickens, and cracked eggs in the womb as demonstrated in (Fig. 5). It was reported that layers, GalliPro MS inclusion improved behavior and made the poultry calmer. In parallel with these observations, laying performance also were enhanced in the trials with probiotic supplementation and reduced the dirty eggs numbers. These findings provide a good indicator of gut health. This reflects a decrease in dysbacteriosis, improved control of pathogenic bacteria and a healthier intestinal microbiome overall [177].

## Multi-Omics Contributions in Optimizing Direct-fed Microbials (DFMS) for Poultry

The genomics combination with the selection and development of DFMs provides precision in handling and meeting the poultry production systems demand and represents a revolutionary approach. Kogut et al. [142] and Wu et al. [159] have demonstrated the significance of genomics in probiotic interventions optimization for enhancing productivity and host health. Hence, genome sequence analysis of microorganisms facilitates the effective utilization of DFMs in poultry. Also, genomics improves the DFMs effectiveness through genomic selection in chickens[176]. Genome sequencing is performed by genome sequencing technologies [178]. Various investigations have proposed the genomics of some DFM strains which are listed in Table 3).

**Table 3:** Some genomic studies of some DFM strains and their effects on poultry

**Examples of Direct-Fed mcrobes**	**Genome sequencing** **platform**	**Sample isolation**	**Effects on the poultry**	**Reference**
***Lactobacillus Salivarius***	Illumina MiSeq platform	Isolation from Chicken gut	↓ shedding of *Salmonella enterica serovar Typhimuriuml*	[179]
***Lactobacillus johnsonii***	Illumina HiSeq or illumina MiSeq platform	Isolation from ileal and cecal	↑ body weight due to influence on weight gain gene in commercial turkeys	[121]
***Bacillus Spp***	PacBio RSII platform	Isolation from the cecal and the fecal of healthy chickens	↓ pathogen resistance by inhibting S*allamonella*	[17]
***Enterococcus Spp***	Illumina Nextseq platform	Isolation from meat chickens (cecal samples)	↑ antimicrobial resistance gene presence	[180]
***Bifidobacterim***	Next-generation sequencing (NGS) platforms	Isolation from different ecosystems	↑ antimicrobial resistance gene presence shows 411 core bifidobacterial genes	[181]

**(↑) increase (↓) decrease**

Genetic markers associated with probiotic efficacy (e.g., stress tolerance or bile salt hydrolase activity) can be identified by genomic tools and these markers are used to accelerate the identification of high-performing strains[182]. As well as in a variety of animal species, GWASs have been used to find the polymorphisms of key genes linked to phenotypic features linked to animal production and disease resistance [125, 183–185].

On the other hand, at community levels, metagenomics enables us to identify microbial abundance, diversity and its roles from functional genomics. Metagenomics provides insights into the taxonomic diversity of the co-metabolizing micro-community within the gastrointestinal tract of poultry. This approach not only facilitates the understanding of nutrient metabolism and the presence of DFMs but also enables the examination of gene-level activities associated with the metabolism of polysaccharides, nitrogen, fatty acids and lipids. Such knowledge can be leveraged to enhance poultry health [186]. Metagenomics can determine the beneficial microorganisms from non-beneficial microorganisms to increase the concentration of beneficial and decrease harmful to control DFMs concentrations in poultry [187].

Moreover, Nutrigenomics allows for outstanding of how nutrients regulate gene and protein expression influencing cellular metabolism [188]. It has been possible to identify specific and reliable biomarkers of the interaction between DFMs and poultry that have implications for poultry growth performance [47, 189]. It has been possible to identify the gene pathways and biological functions that are crucial in regulating the biological effects of DFMs'action on poultry [190]. Additionally, an understanding of the intricacies of the interactions that ultimately determine the outcome of DFMs'effects on poultry as well as the protein–protein interaction (PPI) networks analysis between the poultry and the microbiome can help identify potential flow pathways that could help define the mechanism of action of DFMs on poultry performance and health [191].

## Challenges and Limitations

DFMs utilization in poultry production presents several challenges and limitations. Regulatory frameworks for probiotics in poultry differ across regions, and the approval process can be complex and time-consuming [192–194]. The need to be on the Generally Recognized as Safe (GRAS) list to lower the regulatory obstacles faced during commercialization accounts for a large portion of the restrictions when choosing probiotic cultures [195]. The cost of incorporating DFMs into feed can be a barrier, particularly in large-scale operations with tight profit margins. The economic returns are not always immediate or guaranteed, especially if performance improvements are marginal [196]. Studies sometimes show conflicting results regarding their impact on growth performance and disease resistance, leading to uncertainty among producers as mentioned above. Their sensitivity to pelletizing methods for feed production is one of their limitations along with their susceptibility to environmental influences, the acidity of the stomach, and the bile salts presence in the small intestine [197–201]. Besides, maintaining the viability of live microbial strains during feed processing, storage, and transport can be technically challenging and costly [202]. Moreover, the difficulty in predicting outcomes is due to the complex interactions between poultry genetics, microbiota, and DFMs. Addressing these challenges requires ongoing research to improve strain selection, understand mechanisms, and optimize application methods, ensuring the sustainable and effective use of DFMs in poultry production.

## Future Perspectives

Using DFMs in poultry production is promising, broader and more effective with the revolution in sequencing and genetic modification (GM) technologies. DFMs were genetically engineered to customize probiotics which satisfy our demands [203]. These GM DFMs help in problem-solving due to their stability, substances delivery to mucosal surfaces, reduction of delivery costs and extended shelf life [204, 205]. So, they face the obstacles of traditional probiotics. A *Lactococcus lactis* strain IL1403 was genetically edited to express and manufacture AMPs with a distinguished activity against *E. coli* and *Salmonella* strains. These peptides were expressed by the resulting recombinant *L. lactis* strain, and their effect on the survival and development of *Salmonella* and *E. coli* was examined. Both pathogens were significantly suppressed and the *L. lactis* viability was maintained [206, 207]. As well as GM probiotics increased the stress tolerance of the strain. The GM probiotic strains withstand around 54-fold [207]. It is worth noting that it is necessary to check the GM strains virulence and their pathogenicity, so the safety issues of these stains and their usage are critical concerns [208]. Furthermore, new technologies like synthetic biology and CRISPR-Cas9 may genetically modify probiotics to enhance their functions.

DFMs will also be frequently used with other feed additives i.e. enzymes, phytochemicals, and prebiotics. This combination will make the most of their benefits [209]. Dersjant et al. [209], Nusairat and Wang [210], and Saliu et al. [211] proposed that these synergistic combinations can improve disease resistance, gastrointestinal health, and nutrient absorption. Future studies on genome editing and host-microbe interactions may be the target of ongoing research on poultry production.

## Conclusion of the Effect of Direct-fed Microbials (DFMs) VS Antibiotic Growth Promoters (AGPs) on Poultry Production

DFMs and AGPs have both been widely used to enhance poultry production, but their modes of action and long-term impacts differ significantly. AGPs have traditionally improved growth rates and feed efficiency by suppressing pathogenic bacteria;however, their use raises serious concerns regrading antimicrobial resistance [212–215]. DFMs, on the other hand,offer a natural and sustainable alternative by promoting gut health,improving nutrient absorption,and modulating the immune system [61, 216]. As mentioned before in this review, studies have shown that DFMs can match or even surpass AGPs in enhancing BWG,FCR and resistance to intestinal diseases, particularly when tailored strains are used. Table 4 below summarizes key comparative effects of DFMs and AGPs on poultry production outcomes.

**Table 4:** key comparative effects of DFMs and AGPs on poultry production outcomes

Parameter	Direct-fed microbials (DFMs)	Antibiotic growth promoters (AGPs)
Gut Health	↑ Promotes beneficial microbiota,enhances gut morphology	May impair microbiota balance with prolonged use
Disease Resistance	↑ Enhances immune defense and barrier function	Suppresses pathogens but may lead to resistance buildup
Mortality Rate	↓ Lower mortality in some studies due to immune support	↓ May reduce mortality short-term, but long-term concerns persist
Intestinal Morphology	↑ Increased VH:CD ratio, improved surface area	Neutral or slight improvement, less consistent
Nutrient Absorption	↑ Enhanced through better mucosal integrity and enzyme activity	Indirectly improved via pathogen suppression
Sustainability and Safety	High, no resistance risk, suitable for long-term use	Low, resistance risk, regulatory bans in many regions
Regulatory Status	Widely accepted and encouraged as AGP alternatives	Banned or restricted in many countries
Residues in poultry products	No accumulated harmful residues	Potential of drug residues in products, may cause harm to consumers'health

## Conclusion

DFMs have a positive effect on poultry production. Technology revolution in molecular biology, genome editing, genomics, metagenomics, and bioinformatics facilitate a deep understanding of how DFMs influence poultry health overall and performance. These developments will facilitate and optimize the best microbial and host genotypes for sustainable, safe and high-quality poultry production.

## Data Availability

No datasets were generated or analysed during the current study.

## Abbreviations

DFMs: Direct-fed microbials
CAGR: Compound annual growth rate
LAB: Lactic acid bacteria
GIT: Gastrointestinal tract
SCFAs: Short-chain fatty acids
BWG: Body weight gain
GWAS: Genome-Wide Association Study
SNP: Single nucleotide polymorphism
PLAG1: Pleiomorphic adenoma gene 1
DMRT: Double sex and mab-3-related transcription factor
CASP: Caspase
PTGS2: Prostaglandin-endoperoxide synthase 2
IRF7: Interferon regulatory factor 7
AvBD: Avian ß-defensins
FSs: Transcription factor
NE: Necrotic Enteritis
AMPs: Antimicrobial peptides
TLRs: Toll-like receptors
PRRs: Pattern recognition receptors
MAMPs: Microbial-associated molecular patterns
LPS: Lipopolysaccharides
TGF: Transforming growth factors
IL: Interleukins
IFN: Interferons
TNF: Tumor necrosis factors
TJs: Tight junctions
CLDN: Claudin
ZO-1: Zonula occludens 1
JAM: Junctional Adhesion Molecule
OCLN: Occluding
BMD: Bacitracin methylene disalicylate
GI: Gastrointestinal
CNS: Central nervous system
GABA: γ-Aminobutyric acid
ROS: Reactive oxygen species
PPI: Protein-protein interaction
GRAS: Generally Recognized as Safe
GM: Genetic modification
FCR: Feed conversion rate
VH: Villus height
CD: Crypt depth
AGPs: Antibiotic growth promoters
